# Brigatinib in Japanese patients with tyrosine kinase inhibitor-naive *ALK*-positive non-small cell lung cancer: first results from the phase 2 J-ALTA study

**DOI:** 10.1007/s10147-022-02232-7

**Published:** 2022-08-29

**Authors:** Shunichi Sugawara, Masashi Kondo, Toshihide Yokoyama, Toru Kumagai, Makoto Nishio, Koichi Goto, Kazuhiko Nakagawa, Takashi Seto, Nobuyuki Yamamoto, Kentarou Kudou, Takayuki Asato, Pingkuan Zhang, Yuichiro Ohe

**Affiliations:** 1 Department of Pulmonary Medicine, Sendai Kousei Hospital, Miyagi, Japan; 2 Department of Respiratory Medicine, Fujita Health University School of Medicine, Toyoake, Japan; 3 Department of Respiratory Medicine, Kurashiki Central Hospital, Kurashiki, Japan; 4 Department of Thoracic Oncology, Osaka International Cancer Institute, Osaka, Japan; 5 Department of Thoracic Medical Oncology, The Cancer Institute Hospital of Japanese Foundation for Cancer Research, Tokyo, Japan; 6 Department of Thoracic Oncology, National Cancer Center Hospital East, Kashiwa, Japan; 7 Department of Medical Oncology, Faculty of Medicine, Kindai University, Osaka-Sayama, Japan; 8 Department of Thoracic Oncology, National Hospital Organization Kyushu Cancer Center, Fukuoka, Japan; 9 Internal Medicine III, Wakayama Medical University, Wakayama, Japan; 10 Biostatistics, Japan Development Center, Takeda Pharmaceutical Company Limited, Osaka, Japan; 11 Oncology Clinical Research Department, Oncology Therapeutic Area Unit for Japan and Asia, Takeda Pharmaceutical Company Limited, Osaka, Japan; 12 Takeda Development Center Americas, Inc., Lexington, MA USA; 13 Department of Thoracic Oncology, National Cancer Center Hospital, 5-1-1 Tsukiji Chuo-ku, Tokyo, 104-0045 Japan

**Keywords:** Carcinoma, Non-small cell lung, Anaplastic lymphoma kinase, Tyrosine kinase inhibitor

## Abstract

**Background:**

We evaluated the safety and efficacy of the anaplastic lymphoma kinase (ALK) tyrosine kinase inhibitor (TKI) brigatinib in Japanese patients with TKI-naive *ALK*-positive non-small cell lung cancer (NSCLC) from the phase 2, open-label, single-arm, multicenter J-ALTA study.

**Methods:**

In the TKI-naive cohort of J-ALTA, the primary end point was independent review committee (IRC)-assessed 12-month progression-free survival (PFS). Secondary end points included objective response rate (ORR), intracranial response, overall survival (OS), and safety.

**Results:**

The data were cut approximately 12 months after last patient enrollment. Thirty-two patients with ALK TKI-naive *ALK*-positive NSCLC were enrolled (median age [range], 60.5 [29–85] years; median duration of follow-up, 14.2 [3.2–19.3] months; median treatment duration, 13.8 [0.4–19.3] months). IRC-assessed 12-month PFS was 93.0% (90% confidence interval (CI) 79.2–97.8%); ORR, 96.9% (95% CI 83.8–99.9%), 12-month OS, 96.9% (95% CI 79.8–99.6%), and median OS was not reached. Of five patients with measurable baseline CNS metastases, two had partial intracranial response. The most common treatment-emergent adverse events were increased blood creatine phosphokinase (81%), hypertension (59%), and diarrhea (47%). Grade ≥ 3 adverse events occurred in 91% of patients; pneumonitis was reported in 3 (9%) patients.

**Conclusions:**

In the J-ALTA TKI-naive cohort, brigatinib demonstrated clinically meaningful efficacy consistent with the international phase 3 study. The safety profile in Japanese patients was consistent with previous studies. Brigatinib is an important first-line option for Japanese patients with *ALK*-positive NSCLC.

**Clinical registration:**

NCT03410108

**Supplementary Information:**

The online version contains supplementary material available at 10.1007/s10147-022-02232-7.

## Introduction

Anaplastic lymphoma kinase (*ALK*) gene rearrangements occur in an estimated 3%-5% of patients with non-small cell lung cancer (NSCLC) [1–3]. ALK-targeted therapies that inhibit the ALK tyrosine kinase have been approved for use in NSCLC, including the approved ALK tyrosine kinase inhibitor (TKI) alectinib, which is the current recommended first-line ALK TKI in Japan [4–6]. In the phase 3 J-ALEX study in Japanese patients with ALK TKI-naive NSCLC, alectinib demonstrated longer progression-free survival (PFS) vs crizotinib (median 34.1 vs 10.2 months, hazard ratio [HR], 0.37) [4, 5].

Brigatinib is a next-generation ALK inhibitor with demonstrated central nervous system (CNS) efficacy and activity against clinically relevant *ALK* acquired resistance mutations [7–9]. In the phase 3 ALTA-1L trial (NCT02737501) comparing brigatinib and crizotinib in patients with ALK TKI-naive NSCLC, the primary end point, assessed by blinded independent review committee (BIRC), was met at the first interim analysis [10]. At the final analysis, brigatinib maintained superiority in BIRC-assessed PFS (HR, 0.48; 95% CI 0.35–0.66; 3-year PFS rate: 43% brigatinib vs 19% crizotinib) [11]. Although ALTA-1L was an international trial, it did not include Japanese patients.

The phase 2 J-ALTA trial (NCT03410108), which evaluated the efficacy and safety of brigatinib in Japanese patients with *ALK*-positive NSCLC who were refractory to alectinib or other ALK TKIs, also included a cohort of patients who had not received any previous ALK TKI treatment [12]. Results from the TKI-refractory cohorts of J-ALTA demonstrated that brigatinib has clinically meaningful efficacy in patients previously treated with alectinib [12]. We report here the results from the TKI-naive cohort of J-ALTA.

## Materials and methods

### Study design

**Fig. 1:**
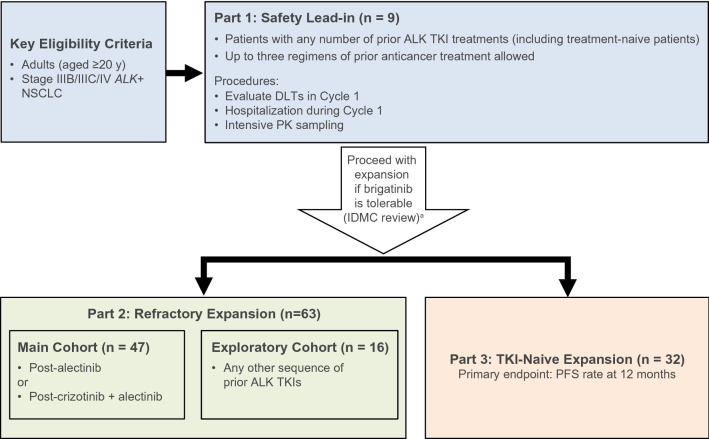
J-ALTA study design. ^a^Although the protocol allowed both TKI-refractory and TKI-naive patients to enroll in the safety evaluation lead-in, all enrolled patients had a history of prior ALK TKI therapy. Thus, the nine patients enrolled in Part 1 were included in the efficacy evaluation of refractory patients (Part 2). *ALK*, anaplastic lymphoma kinase; *ALK +* , anaplastic lymphoma kinase-positive; *DLT*, dose-limiting toxicity; *IDMC*, independent data monitoring committee; *NSCLC*, non-small cell lung cancer; *PFS*, progression-free survival; *PK*, pharmacokinetics; *TKI*, tyrosine kinase inhibitor

J-ALTA was a single-arm, multicenter, phase 2, open-label study of the ALK TKI brigatinib in Japanese patients with *ALK*-positive NSCLC. The trial started with a safety lead-in stage (Part 1) followed by an expansion stage (Parts 2 and 3). Part 2 consisted of two cohorts of patients with *ALK*-positive NSCLC refractory to ALK TKI, one cohort previously treated with alectinib and one previously treated with any ALK TKI, while patients enrolled in Part 3 had no previous TKI therapy (Fig. 1). Results for patients with TKI-refractory *ALK*-positive NSCLC (Part 2) have been previously reported [12]. This manuscript reports the efficacy and safety results from Part 3 of this study.

The study was conducted in compliance with the ethical principles from the Declaration of Helsinki, the International Council for Harmonisation guideline for Good Clinical Practice, and all applicable local regulations. All informed consent and protocol documents were approved by the local institutional review board or ethics committee at each study site. All patients provided informed written consent prior to screening.

### Patients and treatment

Eligible patients were ≥ 20 years of age with histologically or cytologically confirmed stage IIIB, stage IIIC (locally advanced or recurrent and not a candidate for definitive multimodality therapy), or stage IV NSCLC. Patients were required to have documentation of *ALK* rearrangement by a positive result from the Vysis ALK Break Apart fluorescence in situ hybridization (FISH) Probe Kit, the Nichirei Histofine ALK intercalated antibody-enhanced polymer (iAEP) Kit, or the Ventana ALK (D5F3) CDx Assay prior to enrollment and required to submit sufficient tumor tissue for central laboratory testing upon request of study sponsor. Central confirmation of *ALK* rearrangement was not required before enrollment. Patients were also required to have at least one measurable lesion according to the Response Evaluation Criteria in Solid Tumors (RECIST), version 1.1 [13]; had recovered from toxicity related to prior anticancer therapy; had a life expectancy ≥ 3 months; had adequate organ and hematologic function; and had Eastern Cooperative Oncology Group performance status ≤ 2.

Patients were excluded from the Part 3 expansion cohort if they had received any prior TKIs, including ALK inhibitors and vascular endothelial growth factor receptor (VEGFR) inhibitors; received more than one systemic anticancer therapy regimen for locally advanced or metastatic disease; had a history or presence of interstitial lung disease (ILD); had current spinal cord compression; or had symptomatic CNS metastases or asymptomatic CNS metastases requiring an increasing dose of corticosteroids. Patients with asymptomatic leptomeningeal disease without cord compression were permitted. Online Resource 1 lists the complete inclusion and exclusion criteria.

### Procedures

Patients received brigatinib orally at a dose of 90 mg once daily (qd) for the first seven days followed by 180 mg qd (ie, 180 mg qd with 7-day lead-in at 90 mg) and continued treatment until they experienced disease progression or intolerable toxicity, withdrew consent, or discontinued for another reason. Disease was assessed by computed tomography or magnetic resonance imaging scans of the chest, abdomen, pelvis, and brain performed at screening, every two cycles (8 weeks) from Day 1 of Cycle 3 (± seven days) through Day 1 of Cycle 15, every three cycles until the end of therapy, and at the end of treatment if more than four weeks had passed since the last scan.

All radiographic images were assessed by an independent review committee (IRC) according to RECIST version 1.1 [13]. Complete responses (CR) or partial responses (PR) were confirmed at least four weeks after the initial response. Stable disease was evaluated at least 6 weeks after initiation of brigatinib. Patients were followed for survival every 12 weeks after treatment discontinuation.

Adverse events (AEs), including laboratory abnormalities, were categorized using the National Cancer Institute Common Terminology Criteria for Adverse Events version 4.03. An independent data monitoring committee (IDMC) evaluated cases of ILD and pneumonitis, which were reported as serious AEs, and made recommendations as needed.

### End points

The primary end point was PFS rate at 12 months as assessed by an IRC per RECIST version 1.1 [13]. Secondary end points included IRC-assessed confirmed objective response rate (ORR), PFS, duration of response (DoR), disease control rate, and time to response; intracranial PFS (iPFS by IRC); intracranial ORR (iORR by IRC); overall survival (OS); and safety.

### Statistical analysis

A sample size of 32 patients was determined to allow approximately 80% power to rule out the threshold rate of 42.6% (estimated 12-month PFS rate in Kaplan–Meier [KM] plots observed in the ALTA-1L crizotinib arm) when the true 12-month PFS rate is expected to be ≥ 66.5% (estimated 12-month PFS rate in KM plots observed in the ALTA-1L brigatinib arm) with a one-sided alpha of 0.05 [10]. Twelve-month PFS rate and CIs were based on the complementary log–log transformation. This primary analysis was performed at approximately ten months after enrollment of the last patient in the cohort. Summary tabulations with number of observations, mean, standard deviation, median, and minimum and maximum are reported for continuous variables and number and percentage per category for categorical data. KM survival curves are used to report time-to-event data. For secondary end points, statistical inference was performed at a one-sided 0.025 level of significance or a two-sided 0.05 level of significance, as appropriate, to preserve a one-sided overall type I error rate at or below 0.025 or two-sided overall type I error rate at or below 0.05. Statistical analyses were performed using SAS version 9.4.

## Results

### Patients and treatment

**Fig. 2:**
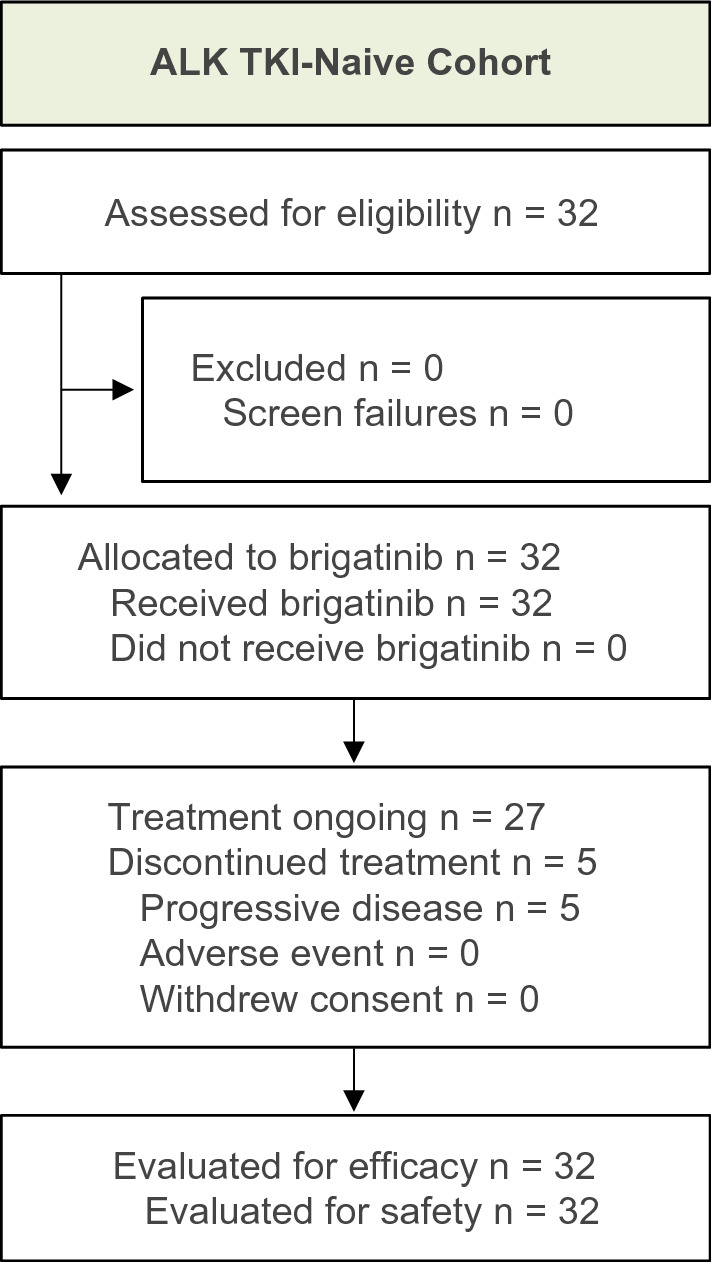
Disposition of patients in the J-ALTA ALK TKI-naive cohort.* ALK*, anaplastic lymphoma kinase; *TKI*, tyrosine kinase inhibitor

Between January 2018 and November 2019, 32 Japanese patients with ALK TKI-naive *ALK*-positive NSCLC were enrolled. The data cutoff of September 29, 2020, was selected so that all enrolled patients could be followed for 12 months. Demographic and baseline characteristics are summarized in Table 1. The median age was 60.5 (range, 29–85) years, and 25% of patients had received prior chemotherapy or checkpoint inhibitor. As of data cutoff, 27 patients (84%) remained on treatment (Fig. 2), with a median duration of follow-up of 14.2 months (range, 3.2–19.3 months). The median treatment duration was 13.8 months (range, 0.4–19.3).

**Table 1:** Baseline patient characteristics

Characteristic	TKI-naive cohort *n* = 32
Age, median (range), y	60.5 (29–85)
Sex, male, *n* (%)	15 (47)
ECOG performance status, *n* (%)	
0	16 (50)
1	15 (47)
2	1 (3)
Smoking history, *n* (%)	
Never smoked	20 (63)
Former smoker	12 (38)
Current smoker	0 (0)
Stage of disease, *n* (%)	
IIIB	0 (0)
IV	32 (100)
Histopathological type of NSCLC, *n* (%)	
Adenocarcinoma	30 (93.8)
Adenosquamous carcinoma	1 (3.1)
Suspected adenocarcinoma	1 (3.1)
Brain metastases at baseline, *n* (%)	7 (22)
Time from initial diagnosis to treatment, median (range), mo	1 (0.3–232)
Methods for *ALK* rearrangement assessment^a^, *n* (%)	
FISH-Vysis	1 (3)
Vysis ALK break apart FISH Probe Kit	13 (41)
Nichirei Histofine ALK iAEP Kit	20 (63)
Ventana ALK (D5F3) CDx Assay	10 (31)
RT-PCR	0 (0.0)
Sequencing	0 (0.0)
Other	3 (9)
Detected fusion partner on ALK, *n* (%)	
EML4	3 (9)
Unknown	29 (91)
Prior chemotherapy, *n* (%)	8 (25)
Prior radiotherapy, *n* (%)	6 (19)
Prior brain radiotherapy, *n* (%)	3 (9)

*ALK* anaplastic lymphoma kinase; *ECOG* Eastern Cooperative Oncology Group; *EML4* echinoderm microtubule-associated protein-like 4; *FISH* fluorescence in situ hybridization; *iAEP* intercalated antibody-enhanced polymer; *NSCLC* non-small cell lung cancer; *RT*–*PCR* reverse transcription polymerase chain reaction; *TKI* tyrosine kinase inhibitor

^a^Patients could have more than one documentation of *ALK* rearrangement detected by different methods

### Primary end point: IRC-assessed progression-free survival

**Fig. 3:**
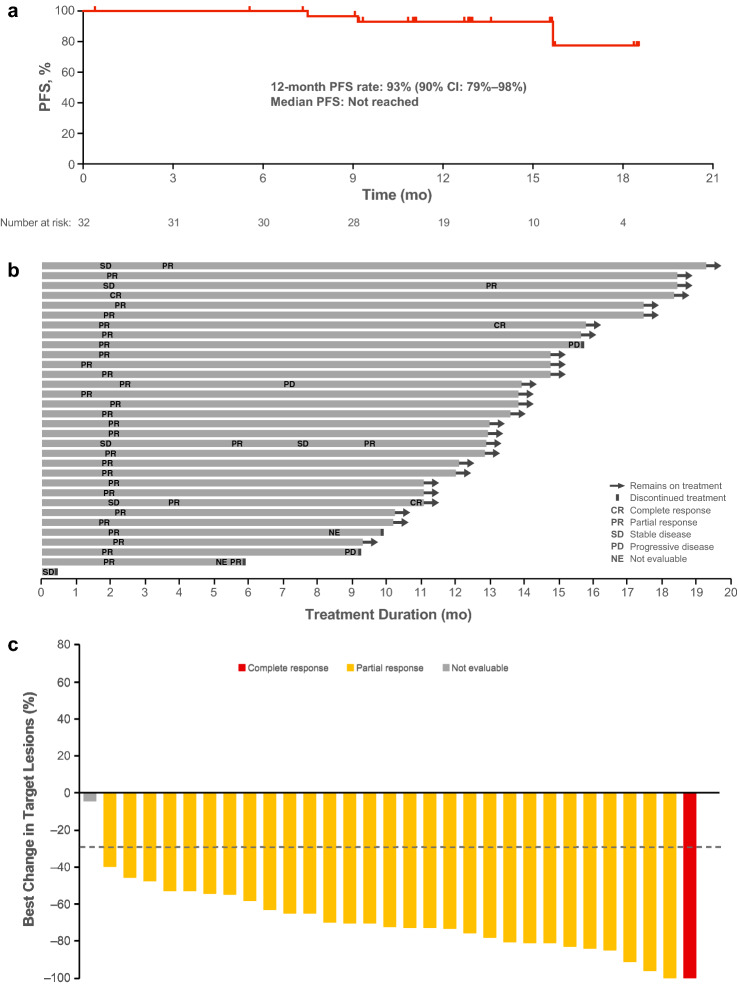

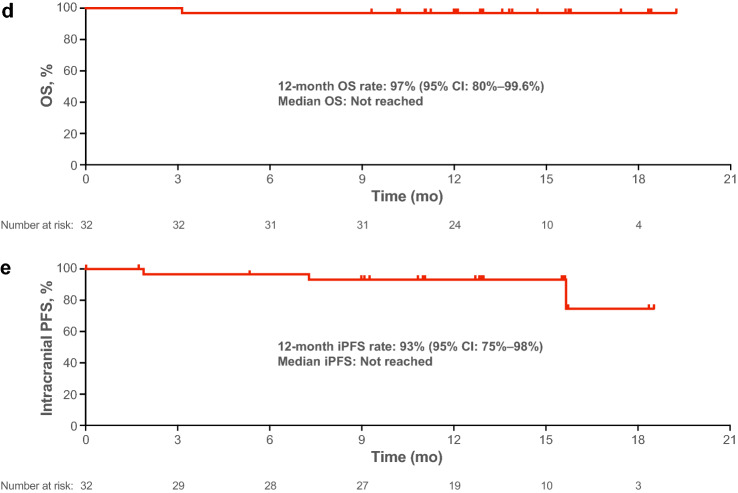
Efficacy of brigatinib in Japanese patients with *ALK*-positive NSCLC not previously treated with ALK TKIs. **a** Kaplan–Meier estimates of IRC-assessed PFS. Of the 32 patients in the cohort, 3 (9.4%) had an event. **b** Objective response per IRC assessments by time on treatment in all patients (*n* = 32). **c** Best percentage change from baseline in the sum of the longest diameters of target lesions per IRC assessment for patients who had a measurable lesion at baseline and at least one postbaseline assessment (*n* = 31). The line at − 30% indicates the threshold for partial response according to RECIST, version 1.1. One patient had investigator-assessed baseline measurable disease and therefore satisfied entry criteria. However, the tumor was located in a nontarget region. Since the IRC review found no measurable lesion in the target region at baseline, the patient was necessarily excluded from the plot. The best overall response in this patient was CR by the IRC and PR by the investigator. SD for ≥ 6 weeks from the first dose could be evaluated as an IRC-assessed SD case. However, one of the 31 responders discontinued treatment due to disease progression (by investigator assessment) prior to being evaluated as IRC-assessed SD, and therefore was not evaluable. **d** Kaplan–Meier estimates of OS. Of the 32 patients in the TKI-naive cohort, one patient died. **e** Intracranial PFS in all patients, regardless of presence of CNS metastases at baseline. Of the 32 patients, 3 (9.4%) had an intracranial event or died. For the analysis of iPFS, systemic disease progression followed by withdrawal from study without intracranial disease progression was censored. Tick marks in Kaplan–Meier plots indicate censored data. *ALK*, anaplastic lymphoma kinase; *CNS*, central nervous system; *iPFS*, intracranial progression-free survival; *IRC*, independent review committee; *NSCLC*, non-small cell lung cancer; *OS*, overall survival; *PFS*, progression-free survival; *RECIST*, Response Evaluation Criteria in Solid Tumors; *SD*, stable disease; *TKI*, tyrosine kinase inhibitor

For the primary end point of IRC-assessed PFS rate at 12 months, 93.0% of patients with TKI-naive *ALK*-positive NSCLC did not have progression or death at 12 months (90% CI 79.2–97.8%) (Fig. 3a). The lower bound of the 90% CI was greater than the threshold PFS rate of 42.6%. Brigatinib showed durable 12-month PFS (90% CI) in subgroup analyses based on age (< 65 years, *n* = 19; 94.1% [73.0‒98.8%]; ≥ 65 years, *n* = 13: 90.9% [61.0‒98.2%]); sex (women, *n* = 17; 93.8% [71.6‒98.8%]; men, *n* = 15; 92.3% [66.1‒98.5%]); presence of CNS metastasis at screening (yes, *n* = 7; 66.7% [27.0–88.2%]; no, n = 25; 100% [100‒100%]); and prior chemotherapy (yes, *n* = 8; 100% [100‒100%]; no, *n* = 24; 90.2% [71.9‒96.8%]).

### Secondary end points

Thirty-one patients responded to treatment, with an IRC-assessed confirmed ORR of 96.9% (95% CI 83.8–99.9%), including two confirmed CRs (6%) and 29 PRs (91%) (Table 2). Investigator-assessed confirmed ORR (93.8% [95% CI 79.2–99.2%]) was consistent with that of the IRC. Figure 3b shows responses over time for all 32 patients in this cohort. Because the treatment decision was made by investigator instead of IRC and there were some inconsistencies in response evaluation between IRC assessment and investigator assessment, one patient continued treatment after progressive disease by IRC, and three patients discontinued treatment before progressive disease by IRC. All three patients had been assessed with progression by investigators before discontinuation. Changes from baseline in target lesions for patients who had confirmed responses are represented in Fig. 3c. One of the 32 patients did not have an IRC-assessed target lesion at baseline and was therefore excluded from the plot. The median best regression in target lesions was − 72%. Among the 31 patients with confirmed response by IRC, median time to response was 1.8 months (range, 0.95–12.9). KM estimated median DoR was not reached (95% CI 13.9–not reached) in patients who had confirmed responses.

**Table 2:** Rates of systemic and intracranial objective response by IRC assessment

	TKI-naive cohort *n* = 32
All patients Confirmed ORR	
*n* (%)	31 (97)
95% CI	83.8–99.9
Best overall response, *n* (%)	
Confirmed complete response	2 (6)
Confirmed partial response	29 (91)
Stable disease	0
Not evaluable	1 (3)
Time to response, months, median (range)	1.8 (0.95, 12.9)
Duration of response, months, median (range)	NR (13.9, NR)
Patients with measurable CNS metastases at baseline	*n* = 5
Confirmed intracranial ORR	
n (%)	2 (40)
95% CI	5.3–85.3
Best overall intracranial response, n (%)	
Confirmed complete response	0
Confirmed partial response	2 (40)
Stable disease	3 (60)
Not evaluable	0

*CNS* central nervous system; *IRC* independent review committee; *NR* not reached; *ORR* objective response rate; *TKI* tyrosine kinase inhibitor

At data cutoff, one patient had died due to disease progression. OS for this cohort at 12 months was 96.9% (95% CI 79.8–99.6%). Median OS was not reached (Fig. 3d).

#### Intracranial efficacy

Among five patients who had measurable CNS metastases at baseline, two patients had IRC-assessed confirmed partial intracranial response; confirmed iORR was 40% (95% CI 5–85%). Among all 32 TKI-naive patients, regardless of baseline CNS metastases status, 12-month iPFS was 93.2% (95% CI 75.5–98.3%; Fig. 3e).

### Safety

Median duration of treatment was 13.8 months (range, 0.4–19.3 months) in the TKI-naive cohort. All patients had one or more treatment-emergent adverse events (TEAEs) (Table 3). The most common (≥ 30%) any-grade TEAEs were increased blood creatine phosphokinase (CPK; 81%), hypertension (59%), diarrhea (47%), increased aspartate aminotransferase (AST; 44%), stomatitis (44%), increased lipase (34%), increased amylase (34%), and increased alanine aminotransferase (ALT; 34%). Among the 32 patients, 14 (44%) had grade ≥ 3 increased blood CPK. Any TEAE of grade 3 or higher occurred in 91% of patients.

**Table 3:** Safety overview and treatment-emergent adverse events of any grade reported in ≥ 10% of all patients or grade ≥ 3 reported in ≥ 2 patients

	TKI-naive cohort, *n* = 32	
	Any	Grade ≥ 3
Overview of adverse events, *n* (%)		
Any adverse event	32 (100)	29 (91)
Adverse event leading to dose reduction	21 (66)	–
Adverse event leading to dose interruption	30 (94)	–
Adverse event leading to discontinuation	0 (0)	–
Adverse events reported in ≥ 10% of patients or grade ≥ 3 reported in ≥ 2 patients, *n* (%)		
Increased blood CPK	26 (81)	14 (44)
Hypertension	19 (59)	11 (34)
Diarrhea	15 (47)	0
Increased AST	14 (44)	2 (6)
Stomatitis	14 (44)	0 (0)
Increased ALT	11 (34)	4 (13)
Increased amylase	11 (34)	3 (9)
Increased lipase	11 (34)	6 (19)
Constipation	8 (25)	0
Pyrexia	8 (25)	0
Muscle spasms	7 (22)	0
Rash	7 (22)	1 (3)
Photosensitivity reaction	6 (19)	1 (3)
Increased blood ALP	5 (16)	0
Nausea	5 (16)	0
Nasopharyngitis	4 (13)	0
Pneumonia	4 (13)	1 (3)
Upper respiratory tract infection	4 (13)	0
Vomiting	4 (13)	0
Hypophosphatemia	3 (9)	2 (6)
Increased GGT	3 (9)	3 (9)

*ALT* alanine aminotransferase; *ALP* alkaline phosphatase; *AST* aspartate aminotransferase; *CPK* creatine phosphokinase; *GGT* gamma-glutamyltransferase; *TKI* tyrosine kinase inhibitor

TEAEs led to study drug interruption in 94% of patients and to dose reduction in 66% of patients. The most common reasons for dose interruption included increases in blood CPK (44%), lipase (19%), amylase (13%), and ALT (13%); hypertension (28%); and pneumonitis (9%). The most common reasons for dose reduction included increased blood CPK (34%) and hypertension (22%).

#### ILD and pneumonitis

Pneumonitis was reported in 3 (9.4%) patients. All cases were reviewed and confirmed by the IDMC. Chest computed tomography showed patterns of faint infiltration/acute hypersensitivity pneumonitis in two patients (days 29 and 223) and cryptogenic organizing pneumonia/chronic eosinophilic pneumonia (day 386) in one patient. All events were grade 1. Following interruption of brigatinib treatment, one case resolved in 12 days, and two cases resolved in 28 days. All patients resumed brigatinib without dose reduction after resolution of pneumonitis (two patients resumed at 90 mg qd and escalated to 180 mg qd since the dose interruption periods were longer than 14 days, per protocol) and have had no recurrence.

## Discussion

This study is the first prospective clinical trial in Japanese patients to evaluate brigatinib efficacy and safety in patients with *ALK*-positive NSCLC who had not received ALK TKI treatment. The predefined primary end point, in which the statistical significance threshold was set as the lower bound of the 90% CI (42.6%), was met with a 12-month PFS of 93.0%. The IRC-assessed confirmed ORR was 96.9% (95% CI 83.8–99.9%) and was largely in agreement with the investigator-assessed confirmed ORR of 93.8% (95% CI 79.2–99.2%). Brigatinib achieved a favorable 12-month iPFS of 93.2% (95% CI 75.5–98.3%; Fig. 3e) despite the inclusion of seven patients with brain metastases at baseline. Based on these results, it would be expected that brigatinib suppresses disease progression in the brain.

With the caveats associated with cross-trial comparisons, these efficacy results are similar to those from other randomized studies of next-generation ALK TKIs in patients with TKI-naive *ALK*-positive NSCLC [4, 10, 14, 15]. The open-label, randomized, phase 3 J-ALEX trial evaluated alectinib versus crizotinib in Japanese patients with ALK inhibitor-naive *ALK*-positive NSCLC. At the first interim analysis, independent review facility (IRF)-assessed median PFS was not reached (95% CI 20.3–not estimable) for alectinib at a median follow-up of 12 months. In patients with at least one measurable lesion, IRF-assessed objective response with alectinib was 92% (76/83 patients; 95% CI 86–98%); investigator-assessed objective response was 85% (88/103 patients; 95% CI 79–92%). OS data were immature, with events reported in only two (2%) of 104 patients [4]. At the first interim analysis of ALTA-1L (median follow-up, 11 months), brigatinib demonstrated BIRC-assessed 12-month PFS of 67% (95% CI 56‒75%) and BIRC-assessed confirmed ORR of 71% (95% CI 62‒78%). BIRC-assessed confirmed iORR was 78% (95% CI 52‒94%) in patients with measurable brain metastases at baseline (*n* = 18) [10]. The ALEX trial of first-line alectinib versus crizotinib in patients with TKI-naive *ALK* + NSCLC reported similar outcomes in the alectinib arm (median follow-up, 19 months). For the primary end point, investigator-assessed PFS, the alectinib 12-month PFS rate was 68.4% (95% CI 61.0‒75.9%) and the investigator-assessed ORR was 82.9% (95% CI 76.0‒88.5%) [14]. In 21 patients with measurable CNS lesions at baseline, IRC-assessed iORR was 81% (95% CI 58–95%) [14]. The phase 3 CROWN trial of lorlatinib versus crizotinib demonstrated comparable efficacy in the lorlatinib arm [15]. In a planned interim analysis, lorlatinib achieved a BIRC-assessed 12-month PFS rate of 78% (95% CI 70‒84%) with 18.3 months’ median follow-up, and BIRC-assessed confirmed ORR was 76% (95% CI 68‒83%). The BIRC-assessed confirmed iORR was 82% (95% CI 57‒96%) in 30 patients with measurable CNS metastases at baseline [15].

As in previous brigatinib studies, the most common AEs in this J-ALTA cohort included elevated CPK, hypertension, elevated amylase, elevated lipase, hepatic enzyme abnormalities, and gastrointestinal AEs. Elevated CPK levels were not associated with clinically meaningful muscle-associated AEs. In an ALTA-1L study subanalysis, safety profiles were similar in Asian and non-Asian patients. However, the Asian patients showed higher rates of AST and ALT elevations compared with these rates in non-Asian patients [16]. Consistent with the ALTA-1L subanalysis, there was a tendency in J-ALTA for a higher incidence of laboratory abnormalities. This trend should be explored further in larger studies, such as real-word evidence studies of Japanese patients. A higher proportion of patients in the TKI-naive cohort of J-ALTA compared with the overall ALTA-1L population had AEs that required dose interruption (94% vs 72%) or dose reduction (66% vs 44%) [11]. Grade ≥ 3 AEs were also higher in the TKI-naive cohort of J-ALTA (91%) compared with ALTA-1L (70%). AEs were manageable, and no treatment discontinuations were required. There were no early-onset pulmonary AEs reported. Three patients had pneumonitis; all events were grade 1 and occurred after day 15 of brigatinib treatment. All cases resolved within 28 days or fewer, and patients resumed brigatinib without dose reduction after resolution of pneumonitis, with no recurrence. In ALTA-1L, early-onset ILD/pneumonitis were reported in 4 of 136 patients (3%) in the brigatinib arm and 1 of 61 patients (2%) who crossed over from crizotinib to brigatinib [17]. No new safety signals were identified.

This study has two limitations. First, J-ALTA was a single-arm trial with limited sample size; 32 patients were ultimately enrolled in this expansion cohort out of 104 total patients enrolled in J-ALTA. Second, while immunohistochemistry was the primary method of *ALK *rearrangement detection at the time when the patients were enrolled, ALK status was obtained in a substantial proportion of patients by FISH-based testing in this study. However, the results presented here provide support for the efficacy and safety of first-line brigatinib already established in the ALTA-1L study.

In the J-ALTA TKI-naive expansion cohort, brigatinib demonstrated clinically meaningful efficacy. The safety profile in Japanese patients was consistent with that in previous studies in other populations, and AEs were manageable. Brigatinib is an important first-line option for Japanese patients with *ALK*-positive NSCLC.

## Supplementary Information

Below is the link to the electronic supplementary material.

Supplementary file1 (PDF 96 KB)

## Data Availability

The data sets, including the redacted study protocol, redacted statistical analysis plan, and individual participant data supporting the results reported in this article, will be made available within 3 months from initial request, to researchers who provide a methodologically sound proposal. The data will be provided after de-identification, in compliance with applicable privacy laws, data protection, and requirements for consent and anonymization.
