# Correction to: Brigatinib in Japanese patients with tyrosine kinase inhibitor-naive *ALK*-positive non-small cell lung cancer: first results from the phase 2 J-ALTA study

**DOI:** 10.1007/s10147-022-02252-3

**Published:** 2022-10-16

**Authors:** Shunichi Sugawara, Masashi Kondo, Toshihide Yokoyama, Toru Kumagai, Makoto Nishio, Koichi Goto, Kazuhiko Nakagawa, Takashi Seto, Nobuyuki Yamamoto, Kentarou Kudou, Takayuki Asato, Pingkuan Zhang, Yuichiro Ohe

**Affiliations:** 1 Department of Pulmonary Medicine, Sendai Kousei Hospital, Miyagi, Japan; 2 Department of Respiratory Medicine, Fujita Health University School of Medicine, Toyoake, Japan; 3 Department of Respiratory Medicine, Kurashiki Central Hospital, Kurashiki, Japan; 4 Department of Thoracic Oncology, Osaka International Cancer Institute, Osaka, Japan; 5 Department of Thoracic Medical Oncology, The Cancer Institute Hospital of Japanese Foundation for Cancer Research, Tokyo, Japan; 6 Department of Thoracic Oncology, National Cancer Center Hospital East, Kashiwa, Japan; 7 Department of Medical Oncology, Faculty of Medicine, Kindai University, Osaka-Sayama, Japan; 8 Department of Thoracic Oncology, National Hospital Organization Kyushu Cancer Center, Fukuoka, Japan; 9 Internal Medicine III, Wakayama Medical University, Wakayama, Japan; 10 Biostatistics, Japan Development Center, Takeda Pharmaceutical Company Limited, Osaka, Japan; 11 Oncology Clinical Research Department, Oncology Therapeutic Area Unit for Japan and Asia, Takeda Pharmaceutical Company Limited, Osaka, Japan; 12 Takeda Development Center Americas, Inc., Lexington, MA USA; 13 Department of Thoracic Oncology, National Cancer Center Hospital, 5-1-1 Tsukiji Chuo-ku, Tokyo, 104-0045 Japan

## Correction to: International Journal of Clinical Oncology 10.1007/s10147-022-02232-7

In the original publication in Table 1, the entry: “Nichirei Histofine RALK iAEP Kit” should read as: “Nichirei Histofine ALK iAEP Kit”. The correct version of Table 1 is given in this correction.

**Table 1:** Baseline patient characteristics

Characteristic	TKI-naive cohort *n* = 32
Age, median (range), y	60.5 (29–85)
Sex, male, *n* (%)	15 (47)
ECOG performance status, *n* (%)	
0	16 (50)
1	15 (47)
2	1 (3)
Smoking history, *n* (%)	
Never smoked	20 (63)
Former smoker	12 (38)
Current smoker	0 (0)
Stage of disease, *n* (%)	
IIIB	0 (0)
IV	32 (100)
Histopathological type of NSCLC, *n* (%)	
Adenocarcinoma	30 (93.8)
Adenosquamous carcinoma	1 (3.1)
Suspected adenocarcinoma	1 (3.1)
Brain metastases at baseline, *n* (%)	7 (22)
Time from initial diagnosis to treatment, median (range), mo	1 (0.3–232)
Methods for *ALK* rearrangement assessment^a^, *n* (%)	
FISH-Vysis	1 (3)
Vysis ALK break apart FISH Probe Kit	13 (41)
Nichirei Histofine ALK iAEP Kit	20 (63)
Ventana ALK (D5F3) CDx Assay	10 (31)
RT-PCR	0 (0.0)
Sequencing	0 (0.0)
Other	3 (9)
Detected fusion partner on ALK, *n* (%)	
EML4	3 (9)
Unknown	29 (91)
Prior chemotherapy, *n* (%)	8 (25)
Prior radiotherapy, *n* (%)	6 (19)
Prior brain radiotherapy, *n* (%)	3 (9)

*ALK* anaplastic lymphoma kinase; *ECOG* Eastern Cooperative Oncology Group; *EML4* echinoderm microtubule-associated protein-like 4; *FISH* fluorescence in situ hybridization; *iAEP* intercalated antibody-enhanced polymer; *NSCLC* non-small cell lung cancer; *RT*–*PCR* reverse transcription polymerase chain reaction; *TKI* tyrosine kinase inhibitor

^a^Patients could have more than one documentation of *ALK* rearrangement detected by different methods

The original publication has been corrected.

