# Correction to: Brigatinib in Japanese patients with tyrosine kinase inhibitor-naive *ALK*-positive non-small cell lung cancer: first results from the phase 2 J-ALTA study

**DOI:** 10.1007/s10147-022-02286-7

**Published:** 2023-01-17

**Authors:** Shunichi Sugawara, Masashi Kondo, Toshihide Yokoyama, Toru Kumagai, Makoto Nishio, Koichi Goto, Kazuhiko Nakagawa, Takashi Seto, Nobuyuki Yamamoto, Kentarou Kudou, Takayuki Asato, Pingkuan Zhang, Yuichiro Ohe

**Affiliations:** 1 Department of Pulmonary Medicine, Sendai Kousei Hospital, Miyagi, Japan; 2 Department of Respiratory Medicine, Fujita Health University School of Medicine, Toyoake, Japan; 3 Department of Respiratory Medicine, Kurashiki Central Hospital, Kurashiki, Japan; 4 Department of Thoracic Oncology, Osaka International Cancer Institute, Osaka, Japan; 5 Department of Thoracic Medical Oncology, The Cancer Institute Hospital of Japanese Foundation for Cancer Research, Tokyo, Japan; 6 Department of Thoracic Oncology, National Cancer Center Hospital East, Kashiwa, Japan; 7 Department of Medical Oncology, Faculty of Medicine, Kindai University, Osaka-Sayama, Japan; 8 Department of Thoracic Oncology, National Hospital Organization Kyushu Cancer Center, Fukuoka, Japan; 9 Internal Medicine III, Wakayama Medical University, Wakayama, Japan; 10 Biostatistics, Japan Development Center, Takeda Pharmaceutical Company Limited, Osaka, Japan; 11 Oncology Clinical Research Department, Oncology Therapeutic Area Unit for Japan and Asia, Takeda Pharmaceutical Company Limited, Osaka, Japan; 12 Takeda Development Center Americas, Inc., Lexington, MA USA; 13 Department of Thoracic Oncology, National Cancer Center Hospital, 5-1-1 Tsukiji Chuo-ku, Tokyo, 104-0045 Japan

**Correction to: International Journal of Clinical Oncology (2022) 27:1828–1838** 10.1007/s10147-022-02232-7

In the original publication, under section: Secondary end points, the sentence starting with: “KM estimated…” should read as:

KM estimated median DoR was not reached (95% CI 13.9–not reached) in patients who had confirmed responses.

In Results section, under heading Secondary end points, the sentence starting with: "Among..." should read as: Among the 31 patients with confirmed response by IRC, median time to response was 1.8 months (range, 0.95–12.9). In Table 2 the value of the entry: Duration of response, months, median (range) has been updated. The revised Table 2 is given in this Correction.

**Table 2:** Rates of systemic and intracranial objective response by IRC assessment

	TKI-naive cohort *n* = 32
All patients Confirmed ORR	
*n* (%)	31 (97)
95% CI	83.8–99.9
Best overall response, *n* (%)	
Confirmed complete response	2 (6)
Confirmed partial response	29 (91)
Stable disease	0
Not evaluable	1 (3)
Time to response, months, median (range)	1.8 (0.95, 12.9)
Duration of response, months, median (range)	NR (13.9, NR)
Patients with measurable CNS metastases at baseline	*n* = 5
Confirmed intracranial ORR	
n (%)	2 (40)
95% CI	5.3–85.3
Best overall intracranial response, n (%)	
Confirmed complete response	0
Confirmed partial response	2 (40)
Stable disease	3 (60)
Not evaluable	0

*CNS* central nervous system; *IRC* independent review committee; *NR* not reached; *ORR* objective response rate; *TKI* tyrosine kinase inhibitor

The original publication has been corrected.

