# Supplementary material for: Brigatinib in Japanese patients with tyrosine kinase inhibitor-naive ALK-positive non-small cell lung cancer: first results from the phase 2 J-ALTA study
Source: Int J Clin Oncol. 2022 Aug 29;27(12):1828–38. doi: 10.1007/s10147-022-02232-7 (PMC9700635; doi:10.1007/s10147-022-02232-7)
Supplement: Supplementary file 1 — Supplementary file1 (PDF 96 KB) [file 10147_2022_2232_MOESM1_ESM.pdf]

Title: Brigatinib in Japanese Patients With Tyrosine Kinase Inhibitor-Naive *ALK*-Positive Non–Small Cell Lung Cancer: First Results From the Phase 2 J-ALTA Study

Authors: Shunichi Sugawara[a], Masashi Kondo[b], Toshihide Yokoyama[c], Toru Kumagai[d], Makoto Nishio[e], Koichi Goto[f], Kazuhiko Nakagawa[g], Takashi Seto[h], Nobuyuki Yamamoto[i], Kentarou Kudou[j], Takayuki Asato[k], Pingkuan Zhang[l], Yuichiro Ohe[m]

[a] Department of Pulmonary Medicine, Sendai Kousei Hospital, Miyagi, Japan, swara357@sendai-kousei-hospital.jp

[b] Department of Respiratory Medicine, Fujita Health University School of Medicine, Toyoake, Japan, mkond@fujita-hu.ac.jp

[c] Department of Respiratory Medicine, Kurashiki Central Hospital, Kurashiki, Japan, ty14401@kchnet.or.jp

[d] Department of Thoracic Oncology, Osaka International Cancer Institute, Osaka, Japan, torukumagai@ybb.ne.jp

[e] Department of Thoracic Medical Oncology, The Cancer Institute Hospital of Japanese Foundation for Cancer Research, Tokyo, Japan, mnishio@jfcr.or.jp

[f] Department of Thoracic Oncology, National Cancer Center Hospital East, Kashiwa, Japan, kgoto@east.ncc.go.jp

[g] Department of Medical Oncology, Kindai University Faculty of Medicine, Osaka-Sayama, Japan, nakagawa@med.kindai.ac.jp

[h] Department of Thoracic Oncology, National Hospital Organization Kyushu Cancer Center, Fukuoka, Japan, setocruise@gmail.com

[i] Internal Medicine III, Wakayama Medical University, Wakayama, Japan, nbyamamo@wakayama-med.ac.jp

[j] Biostatistics, Japan Development Center, Takeda Pharmaceutical Company Limited, Osaka, Japan, kentarou.kudou@takeda.com

[k] Oncology Clinical Research Department, Oncology Therapeutic Area Unit for Japan and Asia, Takeda Pharmaceutical Company Limited, Osaka, Japan, takayuki.asato@takeda.com

[l] Takeda Development Center Americas, Inc., Lexington, MA, USA, Pingkuan.Zhang@takeda.com

[m] Department of Thoracic Oncology, National Cancer Center Hospital, Tokyo, Japan, yohe@ncc.go.jp

Address for correspondence:

Yuichiro Ohe

Department of Thoracic Oncology

National Cancer Center Hospital

5-1-1 Tsukiji Chuo-ku, Tokyo 104-0045, Japan

Phone: 81-3-3542-2511

Fax: 81-3-3545-5270

E-mail: yohe@ncc.go.jp

AUTHOR CONTRIBUTIONS

Study design: Makoto Nishio, Koichi Goto, Kazuhiko Nakagawa, Takashi Seto, Nobuyuki Yamamoto, Kentarou Kudou, Takayuki Asato, Pingkuan Zhang, Yuichiro Ohe; Study investigator: Shunichi Sugawara, Masashi Kondo, Toshihide Yokoyama, Toru Kumagai, Makoto Nishio, Koichi Goto, Kazuhiko Nakagawa, Takashi Seto, Nobuyuki Yamamoto, Yuichiro Ohe; Enrolled patients: Shunichi Sugawara, Masashi Kondo, Toshihide Yokoyama, Toru Kumagai, Makoto Nishio, Kazuhiko Nakagawa, Takashi Seto, Nobuyuki Yamamoto, Yuichiro Ohe; Collection and assembly of data: Takayuki Asato, Kentarou Kudou; Data analysis: Kentarou Kudou; Data interpretation: All authors; Manuscript review and revisions: All authors; Final approval of manuscript: All authors

Online Resource 1

Supplementary Methods

ALK TKI-Naive Patients

Inclusion criteria

1. Male or female Japanese patients aged ≥20 years on the day of consent

2. Have histologically or cytologically confirmed stage IIIB, stage IIIC (locally advanced or recurrent and not a candidate for definitive multimodality therapy), or stage IV NSCLC

3. Documentation of *ALK* rearrangement by a positive result from MHLW Approved tests (eg, Vysis ALK Break Apart FISH [fluorescence in situ hybridization] Probe Kit, the Nichirei Histofine ALK iAEP Kit, or the Ventana ALK [D5F3] CDx Assay) prior to enrollment and required to submit sufficient tumor tissue for central laboratory testing upon request of sponsor. Central confirmation of ALK rearrangement is not required before enrollment

4. Have at least 1 measurable (ie, target) lesion per RECIST version 1.1
   Note: Previously irradiated lesions may not be used for target lesions unless there is unambiguous radiological progression after radiotherapy. Brain lesions may not be used as target lesions if they were 1) previously treated with whole brain radiation therapy (WBRT) within 3 months, or 2) previously treated by stereotactic radiosurgery (SRS) or surgical resection

5. Recovered from toxicities related to prior anticancer therapy to NCI CTCAE version 4.03 Grade ≤1.

6. Have a life expectancy of ≥3 months

7. Have adequate organ and hematologic function, as determined by:

    a. Both alanine aminotransferase (ALT) and aspartate aminotransferase (AST) ≤2.5 times the upper limit of the normal range (ULN) (≤5×ULN is acceptable if liver metastases are present)

    b. Total serum bilirubin ≤1.5×ULN (<3.0×ULN for patients with Gilbert syndrome)

    c. Serum creatinine <1.5×ULN. For patients with creatinine levels above or equal to 1.5×ULN, the patient is eligible if the estimated creatinine clearance using the Cockcroft-Gault formula is ≥30 mL/minute

    d. Serum lipase ≤1.5×ULN and serum amylase ≤1.5×ULN

    e. Absolute neutrophil count (ANC) ≥1.5×10^9/L

    f. Platelet count ≥75×10^9/L

    g. Hemoglobin ≥9 g/dL

    h. Percutaneous oxygen saturation (SpO_2) ≥94% without oxygen support. Patients who need oxygen support are excluded

8. Have an Eastern Cooperative Oncology Group (ECOG) performance status of ≤2

9. Female patients who:

    a. Are postmenopausal for at least 1 year before the screening visit, OR

b. Are surgically sterile, OR

c. If they are of childbearing potential, agree to practice 1 highly effective non-hormonal method of contraception and 1 additional effective (barrier) method at the same time, from the time of signing the informed consent through 4 months after the last dose of study drug, OR

d. Agree to practice true abstinence, when this is in line with the preferred and usual lifestyle of the patient, from the time of signing the informed consent through 4 months after that last dose of study drug. (Periodic abstinence [eg, calendar, ovulation, symptothermal, postovulation methods], withdrawal, spermicides only, and lactational amenorrhea are not acceptable methods of contraception. Female and male condoms should not be used together)

10. Male patients, even if surgically sterilized (ie, status postvasectomy), who:

    a. Agree to practice effective barrier contraception during the entire study treatment period and through 4 months after the last dose of study drug, OR

    b. Agree to practice true abstinence, when this is in line with the preferred and usual lifestyle of the patient, during the entire study treatment period and through 4 months after that last dose of study drug. (Periodic abstinence [eg, calendar, ovulation, symptothermal, postovulation methods], withdrawal, spermicides only, and lactational amenorrhea are

not acceptable methods of contraception. Female and male condoms should not be used together)

Exclusion criteria

1. Received any prior TKI including but not limited to ALK inhibitor and VEGFR TKI
2. Previously received more than 1 regimen (more than 3 regimens in the safety evaluation lead-in part) of systemic anticancer therapy (other than ALK inhibitors) for locally advanced or metastatic disease
   Note: A systemic anticancer therapy regimen will be counted if it is administered over at least 1 cycle. A new anticancer agent used as maintenance therapy will be counted as a new regimen unless it was previously used as initial anticancer therapy. Neoadjuvant or adjuvant systemic anticancer therapy will be counted as a prior regimen if completion of (neo) adjuvant therapy occurred <12 months before the first dose of brigatinib
3. Treatment with any investigational products within 30 days or 5 half-lives of that investigational agent, whichever is longer, before the first dose of brigatinib
4. Received chemotherapy or radiation within 14 days before the first dose of brigatinib, except SRS or stereotactic body radiation therapy
5. Received antineoplastic monoclonal antibodies within 30 days before the first dose of brigatinib

6. Received systemic treatment with strong inhibitors or strong and moderate inducers of cytochrome P450 (CYP) 3A within 7 days before the first dose of brigatinib

7. Had major surgery within 30 days before the first dose of brigatinib. Minor surgical procedures such as venous catheter placement or minimally invasive biopsies are allowed

8. Have been diagnosed with another primary malignancy other than NSCLC, except for the following adequately/definitively treated malignancies: nonmelanoma skin cancer, cervical cancer in situ, nonmetastatic prostate cancer; or patients with another primary malignancy who are definitively relapse-free with at least 3 years elapsed since the diagnosis of the other primary malignancy

9. Have symptomatic CNS metastases (parenchymal or leptomeningeal) at screening or asymptomatic disease requiring an increasing dose of corticosteroids to control symptoms within 7 days before the first dose of brigatinib.

   Note: If a patient has worsening neurological symptoms or signs due to CNS metastasis, the patient needs to complete local therapy and be neurologically stable (with no requirement for an increasing dose of corticosteroids or use of anticonvulsants for symptomatic control) for 7 days before the first dose of brigatinib

10. Have current spinal cord compression (symptomatic or asymptomatic and detected by radiographic imaging). Patients with asymptomatic leptomeningeal disease and without cord compression are allowed

11. Have ongoing or history of interstitial lung disease (ILD) (including interstitial pneumonitis, pneumonitis, radiation pneumonitis, drug-related pneumonitis, organized pneumonia, and pulmonary alveolitis)

12. Have significant, uncontrolled, or active cardiovascular disease, specifically including, but not limited to:

    a. Myocardial infarction within 6 months before the first dose of brigatinib

    b. Unstable angina within 6 months before the first dose of brigatinib

    c. Congestive heart failure within 6 months before the first dose of brigatinib

    d. Uncontrolled atrial arrhythmias despite appropriate medical therapy

    e. History of ventricular arrhythmia, including history of ventricular tachycardia, ventricular fibrillation, or torsade de pointes. Patients with premature ventricular contractions are allowed

    f. Cerebrovascular accident or transient ischemic attack within 6 months before the first dose of brigatinib

13. Have uncontrolled hypertension. Patients with hypertension should be under treatment at the start of screening and demonstrate adequate control of blood pressure

14. Have an ongoing or active infection, including, but not limited to, the requirement for intravenous antibiotics

15. Have a known history of human immunodeficiency virus (HIV) infection. Testing is not required in the absence of history

16. Hepatitis B surface antigen (HBsAg) positive, detectable hepatitis B viral load, or detectable hepatitis C virus (HCV) infection viral load.
    Note: Patients who have positive hepatitis B core antibody (HBcAb) or hepatitis B surface antibody (HBsAb) can be enrolled but must have an undetectable hepatitis B viral load. Patients who have positive HCV antibody can be enrolled but must have an undetectable hepatitis C viral load

17. Have malabsorption syndrome or other gastrointestinal illness that could affect oral absorption of brigatinib

18. Have a known or suspected hypersensitivity to brigatinib or its excipients

19. Female patients who are lactating and breastfeeding or have a positive serum pregnancy test during the screening period
    Note: Female patients who are lactating will be excluded, even if they discontinue breastfeeding

20. Have any condition or illness that, in the opinion of the investigator, would compromise patient safety or interfere with the evaluation of brigatinib
